# Newly diagnosed atrial fibrillation detected by noninvasive screening methods in clinical practice

**DOI:** 10.1016/j.hroo.2025.05.007

**Published:** 2025-05-14

**Authors:** Luis Aguinaga, Walter Bernal, Alejandro Bravo, Raúl Weiss, Leonardo Pilon, Gabriela Reyes, Domingo Pozzer, Alejandro Polti, Daniel Ortigoza, Verónica Tapia, Paola Naput, Sofía Guardiet, Sergio Blanco, Carlos Labadet, Enrique Retyk, Roberto Keegan

**Affiliations:** 1 Centro Internacional de Arritmias, Tucuman, Argentina; 2 Mount Sinai Hospital, Miami, Florida; 3 Instituto Cardiovascular de Rosario, Rosario, Argentina; 4 Instituto de Cardiología de Corrientes, Corrientes, Argentina; 5 Sanatorio Alberdi & Hospital Independencia, Santiago del Estero, Argentina; 6 Hospital Municipal, Moron, Argentina; 7 Hospital Regional de Rio Gallegos, Santa Cruz, Argentina; 8 Sanatorio Delta, Rosario, Argentina; 9 Hospital Interzonal General de Agudos Eva Perón, San Martín, Argentina; 10 Hospital Dr. Cosme Argerich & CEMIC, Buenos Aires, Argentina; 11 Hospital Privado del Sur & Hospital Regional Español, Bahía Blanca, Argentina

**Keywords:** Atrial fibrillation, Newly diagnosed, Screening, Thromboembolic, Bleeding

## Abstract

**Background:**

Patient characteristics and outcomes of newly diagnosed atrial fibrillation (AF) have been investigated in large registries.

**Objective:**

The study aimed to address the role of non-invasive screening tools in diagnosing AF in the Argentinian clinical practice.

**Methods:**

This was an observational retrospective study. Patients’ clinical characteristics and management of newly diagnosed AF detected by a non-invasive screening tool were analyzed.

**Results:**

Of 12,635 AF outpatients, 1018/5947 (17.1%) of newly diagnosed AF were detected by a screening method: electrocardiography monitoring device (82.3%), self-pulse palpation (17.3%), and smartwatch/smartphone (0.4%).

Screening (+) patients were older (73 years [63–81] vs 72 [65–81], *P* = not significant) and had a higher prevalence of ≥ 75 year-old patients (45.5% vs 37.9%, *P* < .001) and women (42.5% vs 41.2%, *P* = not significant).

Adjusted results (odds ratio [95% confidence interval]) by multivariate analysis showed that alcohol consumption (1.74 [1.14–2.65]), thyroid dysfunction (1.26 [1.07–1.49]), coronary artery disease (1.40 [1.19–1.64]), paroxysmal AF (2.39 [1.94–2.96]), asymptomatic condition (2.44 [2.07–2.86]), high risk of thromboembolic and bleeding complications (CHA_2_DS_2_-Vasc, 1.18 [1.06–1.32] and HASBLED, 1.25 [1.08–1.46]), and heart failure (1.23 [1.01–1.49]) were associated with screening (+) patients. Although no difference in the oral anticoagulation therapy was detected, vitamin K antagonists were less frequently prescribed (0.66 [0.53–0.82]). Electrical cardioversion was preferred over class I and III antiarrhythmic drugs (1.33 [1.07–1.66] vs 0.61 [0.44–0.84] and 0.79 [0.64–0.97]).

**Conclusion:**

Non-invasive screening tools are mainly used in Argentinian clinical practice to detect AF in asymptomatic patients at high risk of thromboembolic and bleeding complications. Vitamin K antagonists are less prescribed, and electrical cardioversion is preferred over antiarrhythmic drugs for initial rhythm control.

**Key Findings**

▪ Screening tools have proven to be helpful in increasing the number of newly diagnosed atrial fibrillation cases.
▪ Asymptomatic patients at a high risk of thromboembolic and bleeding complications are the target population to be diagnosed by screening tools in the Argentinian clinical practice.
▪ Vitamin K antagonists are less prescribed, and electrical cardioversion is preferred over antiarrhythmic drugs for initial rhythm control in this group of patients.

## Introduction

Atrial fibrillation (AF) is the most common sustained cardiac arrhythmia in the adult population. The prevalence is estimated to be 2% to 4% and increases with age.^1^ The number of patients with AF is expected to increase considerably in the future as the population ages.^2^^,^^3^

Increased morbidity and mortality are related to an increased risk of myocardial infarction, stroke/systemic embolism, heart failure, dementia, and bleeding. Effective management of risk factors and comorbidities of newly-detected AF can limit the recurrence and progression of arrhythmia and, consequently, prevent clinical manifestations, reduce morbidity and mortality, and reduce health and social care costs.

Non-invasive screening tools and screening strategies can increase AF detection in selected populations. Multiple electrocardiography (ECG)-based and non-ECG-based devices are now available. Routine heart rhythm assessment during health care contact is currently recommended for all individuals aged ≥ 65 years (class IC), and population-based screening via a prolonged non-invasive ECG-based approach should be considered for individuals aged ≥ 75 years or ≥ 65 years with additional CHA_2_DS_2_-Vasc risk factors to ensure earlier detection of AF.^4^

Large registries have addressed the patients’ clinical characteristics and outcomes of newly diagnosed AF.^5^, ^6^, ^7^ Early AF detected by health care screening programs has also been characterized.^8^ This study aimed to analyze the clinical characteristics and management diagnosed by using non-invasive screening tools in patients with newly-detected AF.

## Methods

This retrospective observational study analyzed information from the ASFA (“Argentina Sin Fibrilación Auricular”) project database. Data was collected by the Argentine Federation of Cardiology members invited to voluntarily participate in a study to capture information from outpatients with AF assisted in 2022. Medical records were reviewed, and a web-based form was employed to enter de-identified data. The Centro Internacional de Arritmias (CIAT, Tucuman, Argentina) was the coordinating center. Patients first diagnosed during the study period were considered to have newly-detected AF and were included in the study. The diagnosis was confirmed by a 12-lead ECG or an ECG strip with arrhythmia lasting ≥ 30 seconds in all patients. The clinical characteristics, diagnostic methods, oral anticoagulation therapy (OAC), and rate and rhythm control therapies were analyzed. Patients with and without non-invasive screening tools contributing to the AF diagnosis were compared (groups screening + and screening +, respectively). The research reported in this paper adhered to the STROBE guidelines, and the study was approved by the CIAT institutional review board. According to this and the protocol's design, which included patient information de-identification, no additional institutional approval or patient consent was required.

### Statistical analysis

Categorical variables are reported as absolute frequencies and percentages, and quantitative variables are reported as the means ± SDs or medians and quartiles (Q1–Q3). The Pearson χ2 test was used to compare categorical variables, and the Mann-Whitney *U* test was used for quantitative variables. After the univariate analysis, any covariate with a *P* < .1 was entered in a multivariate logistic regression model to eliminate potential confounders. A 2-tailed *P* < .05 was considered statistically significant. SPSS v.26 was used for the analysis.

## Results

**Figure 1:**
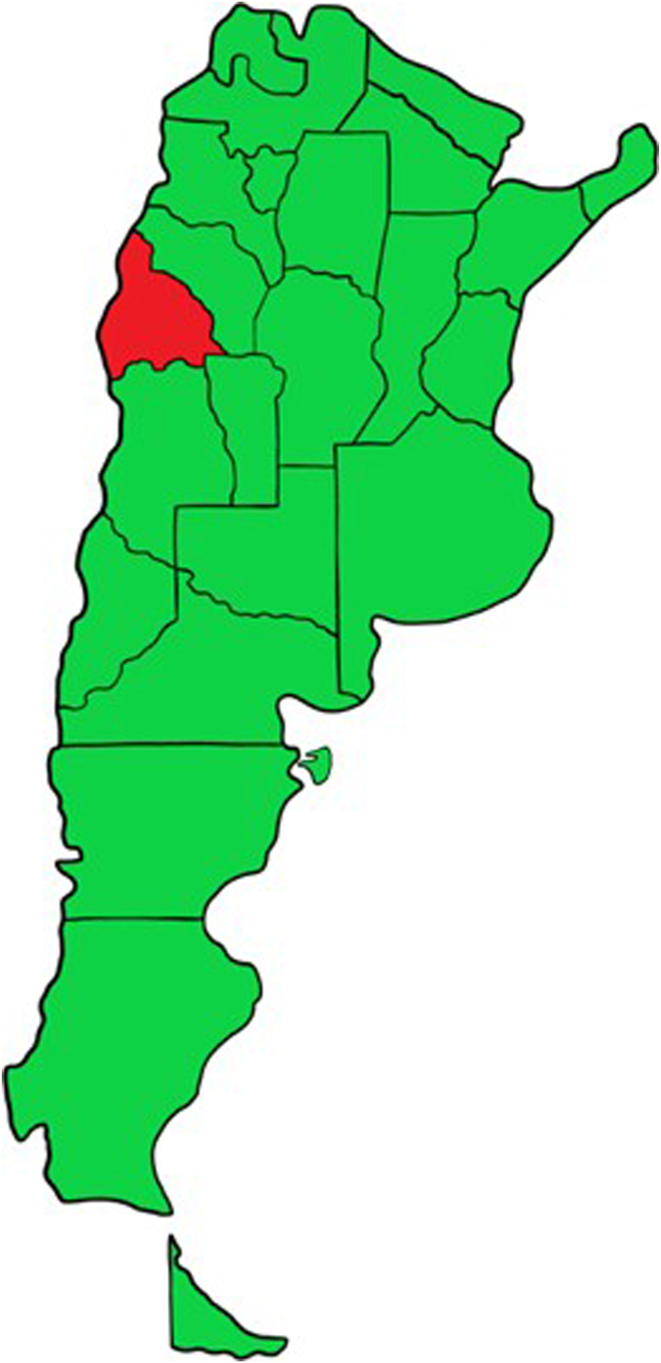
Argentinian provinces represented in the ASFA database (green). ASFA = Argentina Sin Fibrilación Auricular.

Data from 12,635 outpatients with AF were collected by 132 cardiologists from 21 of 24 (96%) Argentinian provinces. (Figure 1, Supplemental Appendix). Newly diagnosed patients with AF were selected for inclusion in the analysis (5947, 47.0%). A non-invasive screening method contributing to the AF diagnosis was identified in 1018 patients (17.1%). The most common tool was an ECG monitoring device (82.3%), followed by self-pulse palpation (17.3%) and a smartwatch/smartphone (0.4%) (Table 1). Table 2 shows data on newly diagnosed AF in the total population and the comparison between the groups. The median age was 72 years (65–81), 39.2% were ≥ 75 years, and 24.9% were < 65 years old.Women represented 41.4% of the total population. Hypertension was present in 75.8% of the patients, diabetes in 26.2%, and dyslipidemia in 46.2%. Smoking status and current smoking status were detected in 33.2% and 10.7% of the patients, respectively. Almost 2 of 3 of the patients (65.2%) had a sedentary lifestyle. Alcohol consumption (heavy drinking according to the National Institute on Alcohol Abuse and Alcoholism definition) was reported by 2.7% of the patients. Thyroid dysfunction, impaired kidney function, peripheral vascular diseases, and lung diseases were present in 31.6%, 8%, 18.9%, and 14.1% of the patients, respectively. Previous coronavirus disease 2019 (COVID-19) infection was reported by 43.7% of patients, and COVID-19 vaccination by 74.2%. Structural heart disease was present in at least 47.2% of the patients (2806/5947), with CAD being the most prevalent (27.2%). Chagas seropositivity was detected in 14.3% of the patients, and a cardiac implantable electronic device (CIED) was present in 17.9%. Asymptomatic disease was the clinical presentation in 55.4% of the patients. Paroxysmal AF was the most common AF pattern (75.7%). Most patients had intermediate or high thromboembolic risk scores (CHA_2_DS_2_-Vasc score of 1 or ≥ 2; 23.2% and 68.5%, respectively) and moderate or high bleeding risk scores (HASBLED score of 1–2 or ≥3; 58.1% and 19.0%, respectively). OAC was initiated in 71.1% of the patients, most frequently direct OACs (DOACs) (52.2%). A beta-blocker agent was indicated in 37.7% of the cases, and a class III antiarrhythmic drug (AAD) in 29.6%. Electrical cardioversion and catheter ablation were performed in 11.3% and 20.7% of patients, respectively. Heart failure was present in 26.0%, and stroke and bleeding in 2.8% and 5.1% of the patients, respectively.

**Table 1:** Screening tools contributing to the diagnosis of newly detected AF

Diagnosis	Number	Percentage
Total	12,635	
Newly diagnosed AF	5,947	47.0%
Screening tool	1,018	17.1%
ECG monitoring	838	82.3%
Self-pulse palpation	176	17.3%
Smartwatch/Smartphone	4	0.4%

AF = atrial fibrillation; ECG = electrocardiography.

**Table 2:** Newly detected AF diagnosed with (+) and without (−) a screening tool

	Total		Screening (+)		Screening (−)		Dif.	*P* value	OR (95% CI)
Total	5947		1018		4929				
Age	72 [65–81]		73 [63–81]		72 [65–81]		1.0	.115	
<65	1480	24.9%	266	26.1%	1214	24.60%	1.5%	.314	1.08 (0.93–1.26)
65–74	2135	35.9%	289	28.4%	1846	37.50%	-9.1%	<.001	0.66 (0.57–0.77)
≥75	2332	39.2%	463	45.5%	1869	37.90%	7.6%	<.001	1.37 (1.19–1.57)
Gender (female)	2464	41.4%	433	42.5%	2031	41.2%	1.3%	.433	1.06 (0.92–1.21)
CV risk factors									
Hypertension	4506	75.8%	702	69.0%	3804	77.2%	-8.2%	<.001	0.66 (0.57–0.76)
Diabetes	1561	26.2%	238	23.4%	1323	26.8%	-3.5%	.022	0.83 (0.71–0.97)
Dyslipidemia	2749	46.2%	468	46.0%	2281	46.3%	-0.3%	.859	0.99 (0.86–1.13)
Smoking	1972	33.2%	341	33.5%	1631	33.1%	0.4%	.802	1.02 (0.88–1.18)
Current smoker	639	10.7%	119	11.7%	520	10.5%	1.1%	.285	1.12 (0.91–1.39)
Sedentary lifestyle	3875	65.2%	522	51.3%	3353	68.0%	-16.7%	<.001	0.49 (0.43–0.57)
Comorbidities									
Alcohol consumption	163	2.7%	42	4.1%	121	2.5%	1.7%	.003	1.71 (1.20–2.45)
Thyroid dysfunction	1882	31.6%	401	39.4%	1481	30.0%	9.3%	<.001	1.51 (1.32–1.74)
Impaired kidney function	478	8.0%	93	9.1%	385	7.8%	1.3%	.157	1.19 (0.94–1.50)
Peripheral vascular disease	1126	18.9%	198	19.4%	928	18.8%	0.6%	.644	1.04 (0.88–1.23)
Lung disease	840	14.1%	96	9.4%	744	15.1%	-5.7%	<.001	0.59 (0.47–0.73)
COVID-19 infection	2600	43.7%	380	37.3%	2220	45.0%	-7.7%	<.001	0.73 (0.63–0.84)
COVID-19 vaccination	4415	74.2%	651	63.9%	3764	76.4%	-12.4%	<.001	0.55 (0.48–0.63)
CAD	1618	27.2%	342	33.6%	1276	25.9%	7.7%	<.001	1.45 (1.25–1.67)
Valvular heart disease	742	12.5%	121	11.9%	621	12.6%	-0.7%	.531	0.94 (0.76–1.15)
Dilated cardiomyopathy	795	13.4%	142	13.9%	653	13.2%	0.7%	.550	1.06 (0.87–1.29)
Hypertrophic cardiomyopathy	200	3.4%	26	2.6%	174	3.5%	-1.0%	.116	0.72 (0.47–1.09)
Chagas’ seropositive test	851	14.3%	125	12.3%	726	14.7%	-2.5%	.042	0.81 (0.66–0.99)
CIED	1065	17.9%	174	17.1%	891	18.1%	-1.0%	.456	0.93 (0.78–1.12)
Paroxysmal AF	4499	75.7%	834	81.9%	3665	74.4%	7.6%	<.001	1.56 (1.32–1.86)
Asymptomatic patient	3295	55.4%	708	69.5%	2587	52.5%	17.1%	<.001	2.07 (1.79–2.39)
CHA2DS2-Vasc	2.4 ± 1.5		2.7 ± 1.6		2.3 ± 1.5			<.001	
Low	496	8.3%	88	8.6%	408	8.3%	0.4%	.700	1.05 (0.82–1.33)
Intermediate	1380	23.2%	158	15.5%	1222	24.8%	-9.3%	<.001	0.56 (0.46–0.67)
High	4071	68.5%	772	75.8%	3299	66.9%	8.9%	<.001	1.55 (1.33–1.81)
HASBLED	1.5 ± 1.3		1.9 ± 1.5		1.4 ± 1.2			<.001	
Low	1362	22.9%	211	20.7%	1151	23.4%	-2.6%	.070	0.86 (0.73–1.01)
Moderate	3457	58.1%	489	48.0%	2968	60.2%	-12.2%	<.001	0.61 (0.53–0.70)
High	1128	19.0%	318	31.2%	810	16.4%	14.8%	<.001	2.31 (1.98–2.69)
Antithrombotic therapy	5075	85.3%	844	82.9%	4231	85.8%	-2.9%	.016	0.80 (0.67–0.96)
OAC	4226	71.1%	670	65.8%	3556	72.1%	-6.3%	.016	0.74 (0.64–0.86)
VKA	1119	18.8%	148	14.5%	971	19.7%	-5.2%	<.001	0.69 (0.57–0.84)
DOAC	3107	52.2%	522	51.3%	2585	52.4%	-1.2%	.497	0.95 (0.83–1.09)
Rivaroxaban	2296	38.6%	386	37.9%	1910	38.8%	-0.8%	.619	0.97 (0.84–1.11)
Dabigatran	357	6.0%	67	6.6%	290	5.9%	0.7%	.393	1.13 (0.86–1.48)
Apixaban	454	7.6%	69	6.8%	385	7.8%	-1.0%	.259	0.86 (0.66–1.12)
Antiplatelets	2952	49.6%	517	50.8%	2435	49.4%	1.4%	.421	1.06 (0.92–1.21)
Rate control drugs									
Beta blockers	2240	37.7%	410	40.3%	1830	37.1%	3.1%	.059	1.14 (0.99–1.31)
Calcium antagonists	154	2.6%	41	4.0%	113	2.3%	1.7%	.002	1.79 (1.24–2.57)
AADs	2285	38.4%	325	31.9%	1960	39.8%	-7.8%	<.001	0.71 (0.62–0.82)
Class I	524	8.8%	68	6.7%	456	9.3%	-2.6%	.008	0.70 (0.54–0.91)
Class III	1761	29.6%	257	25.2%	1504	30.5%	-5.3%	<.001	0.77 (0.66–0.90)
Electrical cardioversion	672	11.3%	152	14.9%	520	10.5%	4.4%	<.001	1.49 (1.22–1.81)
Catheter ablation	1234	20.7%	224	22.0%	1010	20.5%	1.5%	.278	1.09 (0.93–1.29)
Clinical outcomes									
Heart failure	1544	26.0%	304	29.9%	1240	25.2%	4.7%	.002	1.27 (1.09–1.47)
Stroke	165	2.8%	40	3.9%	125	2.5%	1.4%	.014	1.57 (1.09–2.26)
Bleeding	302	5.1%	65	6.4%	237	4.8%	1.6%	.037	1.35 (1.02–1.79)

AAD = antiarrhythmic drug; AF = atrial fibrillation; CAD = coronary artery disease; CHA_2_DS_2_-Vasc = congestive heart failure, hypertension, age (2 points 75 years or older), diabetes mellitus, stroke/transient ischemic attack (2 points), vascular disease; CI = confidence interval; CIED = cardiac implantable electronic device; COVID-19 = coronavirus disease 2019; CV = cardiovascular; Dif. = differentiation; DOAC = direct oral anticoagulant; HASBLED = hypertension, abnormal renal or liver function, stroke, bleeding, labile INR, elderly, drugs and alcohol; OAC = oral anticoagulation therapy; OR = odds ratio; VKA = vitamin K antagonist.

Patients in the screening (+) group were older than those in the screening (-) group, but the difference was not statistically significant (73 [63–81] vs 72 [65–81]). However, significant differences in age groups were observed: patients ≥ 75 years were significantly more frequent (differentiation 7.6%) and those aged 65–74 years significantly less (differentiation −9.1%). No significant difference in female gender was observed between the groups (42.5% vs 41.2%). Compared with the screening (−) group, the screening (+) patients presented a lower prevalence of most cardiovascular risk factors, with significant differences in hypertension, diabetes, and a sedentary lifestyle. Smoking and current smokers were more common, but the differences were not significant. Alcohol consumption, thyroid dysfunction, and CAD were significantly more prevalent. Lung disease, COVID-19 infection, COVID-19 vaccination, and Chagas seropositive tests were significantly less frequent. Although there was a higher prevalence of impaired kidney function, peripheral vascular disease, and dilated cardiomyopathy, the differences were not statistically significant. A lower frequency of valvular heart disease and hypertrophic cardiomyopathy was detected, but the difference was not significant. CIED was also found less frequently, but the difference was not significant. Asymptomatic clinical presentation was significantly more prevalent. The paroxysmal pattern was also significantly more common. Thromboembolic and bleeding risk scores (CHA_2_SDS_2_-VASc and HASBLED) were significantly higher. A significantly lower number of OAC prescriptions was driven by a significantly lower vitamin K antagonist (VKA) usage. DOACs were also less prescribed, but the differences were not significant. class I and class III AADs were significantly less indicated. Electrical cardioversion was performed significantly more frequently. Although catheter ablation therapy was more common, the difference was not statistically significant. The clinical variables associated with the outcomes (heart failure, stroke, and bleeding) were significantly more prevalent in this screening (+) patients.

Table 3 shows the results adjusted by the multivariate analysis. Alcohol consumption, thyroid dysfunction, CAD, paroxysmal pattern of the arrhythmia, no previous symptoms, high risk of thromboembolic and bleeding complications (CHA_2_DS_2_-Vasc and HASBLED), and heart failure were clinical characteristics associated with these patients. On the contrary, age 65–74 years, hypertension, diabetes, sedentary lifestyle, lung disease, and COVID-19 vaccination were associated with a lower probability of being associated with this population. Electrical cardioversion was more likely to be performed in these patients, and AADs and VKAs were less likely to be prescribed.

**Table 3:** Multivariate analysis

Variable	*P* value	OR (95% CI)
Asymptomatic	<.001	2.44 (2.07–2.86)
Paroxysmal	<.001	2.39 (1.94–2.96)
Alcohol consumption	.011	1.74 (1.14–2.65)
CAD	<.001	1.40 (1.19–1.64)
Electrical cardioversion	.011	1.33 (1.07–1.66)
Thyroid dysfunction	.006	1.26 (1.07–1.49)
HASBLED	.004	1.25 (1.08–1.46)
Heart failure	.036	1.23 (1.01–1.49)
CHA2DS2-Vasc	.003	1.18 (1.06–1.32)
Diabetes	.013	0.80 (0.67–0.95)
AAD class III	.026	0.79 (0.64–0.97)
Age 65–74	.004	0.77 (0.64–0.92)
Lung disease	.022	0.74 (0.57–0.96)
VKA	<.001	0.66 (0.53–0.82)
Sedentary lifestyle	<.001	0.63 (0.54–0.74)
Hypertension	<.001	0.62 (0.51–0.75)
COVID-19 vaccination	<.001	0.62 (0.51–0.75)
AAD class I	.002	0.61 (0.44–0.84)

AAD = antiarrhythmic drug; CAD = coronary artery disease; CHA2DS2-Vasc = congestive heart failure, hypertension, age (2 points 75 years or older), diabetes mellitus, stroke/transient ischemic attack (2 points), vascular disease; CI = confidence interval; COVID-19 = coronavirus disease 2019; HASBLED = hypertension, abnormal renal or liver function, stroke, bleeding, labile INR, elderly, drugs and alcohol; OR = odds ratio; VKA = vitamin K antagonist.

## Discussion

Early AF detection is crucial to reducing all complications associated with this arrhythmia: the increased risk of myocardial infarction, stroke, heart failure, dementia, cognitive decline, mortality, and increased social and health care costs.^9^ Early AF diagnosis in symptomatic patients and screening focused on asymptomatic individuals can increase AF detection.

Large global registries have addressed the patient clinical characteristics and outcomes of newly-detected AF. In the Global Registry on Long-Term Oral Antithrombotic Treatment in Patients with Atrial Fibrillation (GLORIA-AF), the median age and female gender proportion of 15,092 patients at risk for stroke were similar to those in our study (71 [64–78] vs 72 [65–81] years; 45.5% vs 41.4%, respectively). The prevalences of hypertension, diabetes, dyslipidemia, and CAD were also similar (74.6% vs 75.8%; 23.1% vs 26.2%; 39.9% vs 46.2%; 20.3% vs 27.2%). A lower frequency of abnormal kidney function was observed (1.6% vs 8.0%). The prevalence of the paroxysmal pattern was similar to that reported in our study (53.4% vs 55.4%). A notable difference in thromboembolic risk was observed. A high CHA_2_DS_2_-Vasc risk score was more common in the GLORIA-AF cohort (86.1% vs 68.5%). This difference can be attributed to the fact that patients with at least an intermediate thromboembolic risk score (CHA_2_DS_2_-Vasc ≥ 1) were an inclusion criterion in GLORIA-AF. Another major difference was the lower bleeding risk in these patients than in our study (HASBLED high-risk score 9.1% vs 19.0%). Antithrombotic therapy varied according to the region in the GLORIA-AF study. The proportion of patients receiving antithrombotic therapy (OAC and/or antiplatelets) and OAC was lower in our study than in the Latin American and Argentinian populations in the GLORIA-AF registry (85.3% vs 95.8% and 97.4%; and 71.1% vs 85.3% and 88.2%, respectively). This finding could be related to the previously mentioned increased risk for stroke in the patients included in the GLORIA-AF study.

The Global Anticoagulant Registry in the Field-Atrial Fibrillation (GARFIELD-AF) data of 17,162 patients with newly diagnosed AF who were prospectively enrolled between 2010 and 2013 and recruited from 858 sites in 30 countries were similar to those of GLORIA-AF. The mean age was 69.8 ± 11.4 years, 43.8% were women, 78.1% had hypertension, 21.9% had diabetes, 40.1% had hypercholesterolemia, and 19.9% had CAD. The thromboembolic risk profile was similar to that of the GLORIA-AF study (CHA_2_DS_2_-Vasc low, intermediate, and high risk: 2.3%, 11.8%, and 85.9%, respectively), but the bleeding risk was higher compared with the GLORIA-AF and lower compared with our patients (HASBLED high-risk score 13.1%).^10^

Newly-detected AF patient clinical characteristics in health care screening programs were analyzed in a recent retrospective multicenter registry, including 3318 newly diagnosed AF cases in an outpatient clinic in Japan.^8^ Compared with non-screening patients (2489, 75%), patients detected by the regular health screening program (829, 25%) presented a lower thromboembolic risk (CHADS2 scores 1.01 vs 1.50, *P* < .001), a higher prevalence of persistent AF (odds ratio [95% confidence interval] 2.21 [1.88–2.60]), more frequent asymptomatic presentation (odds ratio [95% confidence interval] 3.19, [2.71–3.76]), and better baseline quality-of-life scores (83.6 vs 75.0; *P* < .001). Catheter ablation was more frequently performed in the health screening group at follow-up (44.4% vs 34.1%; *P* < .001).

The performance of different alternative methods to detect AF other than a conventional 12-lead ECG has been evaluated. In the Apple Heart Study, more than 410,000 healthy subjects who use their Apple Watch® were enrolled. An irregular pulse notification was observed in 0.52% of the entire population, and this number increased when the subject was older or had a higher CHA_2_DS_2_-Vasc score.^11^ The positive predictive value of such an irregular pulse notification to represent true AF was high (84%). The rates of detection of atrial arrhythmias via a 30-day external monitor and an implantable cardiac monitor were significantly higher than those via a 24-hour Holter monitor.^12^^,^^13^ The sensitivity, specificity, and predictive value must be considered when evaluating the diagnostic performance of methods for detecting AF. Low sensitivity, specificity, and positive predictive values have been reported for pulse taking by a nurse in patients ≥ 65 years seeking care for any reason at primary centers (56%, 81%, and 7%, respectively).^14^ The diagnostic yield of different ECG monitoring tools is variable; 0.5% for intermittent handled ECGs,^15^ between 4% and 16% for continuous external ECGs,^12^^,^^16^ and 34%–35% for implantable loop recorders.^17^^,^^18^ CIED performance depends on the duration criteria of the atrial heart rate episode. These were present in 45% of patients when the duration criterion was >6 min, but in 24% when the duration criterion was > 24 hours.^19^ Regular interrogation of the devices is strongly recommended (class I) to increase the detection of AF.^20^

In our study, at least 1 screening tool (ECG monitoring, self-pulse palpation, or smartwatch/smartphone) was employed to diagnose 17.1% of newly-detected AF cases. Our data show that these patients differed from those described by large registries and health care screening programs.^5^, ^6^, ^7^ The probability of an Argentinian patient being diagnosed by screening tools is higher when alcohol consumption, thyroid dysfunction, CAD, paroxysmal pattern of the arrhythmia, no previous symptoms, high risk of thromboembolic and bleeding complications, and/or heart failure are present. The reasons why electrical cardioversion was more commonly performed and AADs were less frequently used are unclear. A potential explanation is that the higher prevalence of asymptomatic patients could favor this therapy over catheter ablation as the first option for rhythm control strategy. The high bleeding risk profile could affect physicians' decisions to prescribe fewer VKAs, according to the evidence supporting that major bleeding complications are lower with DOACs compared with VKAs.

## Limitations

This study has potential limitations. The retrospective observational nature could introduce selection bias, and data collection from medical records could lead to inconsistencies. Although a global consensus to guide AF management is available, differences in patient characteristics, physicians’ preferences, health care systems, and diagnostic and therapeutic resource availability can limit the extrapolation of these results to other regions worldwide.

## Conclusion

Several detection tools are available to identify early AF. Large studies have characterized newly diagnosed AF detected by non-screening and screening strategies. In Argentinian clinical practice, asymptomatic patients at high risk of thromboembolic and bleeding events are the target population for detecting AF by these screening methods. Electrical cardioversion is preferred over AADs for the initial rhythm control, and VKAs are less prescribed in this population.
