# Supplementary material for: Newly diagnosed atrial fibrillation detected by noninvasive screening methods in clinical practice
Source: Heart Rhythm O2. 2025 May 14;6(8):1212–7. doi: 10.1016/j.hroo.2025.05.007 (PMC12411954; doi:10.1016/j.hroo.2025.05.007)
Supplement: Appendix [file mmc1.docx]

# Appendix: Provinces and Investigators

Provinces (#investigators): *Buenos Aires city and Buenos Aires Province (38), Catamarca (1), Chaco (7), Chubut (1), Córdoba (9), Corrientes (5), Entre Rios (5), Formosa (3), Jujuy (4), La Pampa (1), La Rioja (3), Mendoza (3), Misiones (1), Neuquen (1), Río Negro (2), Salta (6), San Luis (4), Santa Cruz (1), Santa Fe (16), Santiago del Estero (5), Tiera del Fuego (1), Tucumán (15)*

Investigators: *Agliano Bruno; Aguero Clemente; Aguinaga Luis; Alainez Federico; Albisu Juan; Alegrini Eduardo; Alfie Claudio; Almiron Yesica; Alterini Pablo; Alvarez Serafina; Amoroso Alejandro; Arabia Luis; Aramayo Carolina; Barrios Perla; Benavente Claudio; Benito Rocio; Bernal Walter; Blanco Sergio; Bravo Alejandro; Bustamante Gustavo; Cabrera Gaston; Cagnolatti Alfredo; Campos Marcelo; Cardona Mario; Casanova Pablo; Castelli Alejandro; Chelle Edelmiro; Chlawit Marwan; Chong Velazquez Paola ; Colombo Hugo; Corbo Alejandro; Costa Gonzalo; Costa Nestor ; Courtade Paola; Courtade Valeria; Damascena Juliane; Davila Silvia; Diangelo Silvano; Dorcasberro Luis; Fares Beder; Ferrer Ricardo; Flores Roberto; Franchi Marcelo; Freijo Luis; Fuentes Analia; Galarza Nadia; García Angel; Garcia Vasquez Fortunato; Garofalo Pablo; Gomez Romina; Gonzalez Paola; Goyeneche Emilia; Gregorietti Franco; Guardiet Sofia; Guerrero Maria; Guimaraenz Marcelo; Guzman Luis; Hasbani Valeria; Hernandez Daniel; Idolo Nestor; Inclan Sofia; Iralde Gustavo; Jove Agustina; Justiniano Andrea; Labadet Carlos; Lanzotti Marcelo; Lopez Alejandro; Lopez Lorena; Lopez Santi Maria; Manzolillo Hector; Manzzardo Juan; Martinez Griffa Fabian; Martinez Griffa Juan; Massa Maximiliano; Mazo Guillermo; Mendoza Carlos; Meretta Augusto; Meta Javier; Montenegro Fernando; Moreno Alejandro; Moris Cecilia; Naput Paola; Nuñez Aida; Ochoa Miguel; Ortigoza Daniel; Ortiz Patricia; Pantich Rolando; Parucci Ayelen; Peñafort Fernando; Pereira Gustavo; Pereira Luciano; Perez Antonio; Perez Graciela; Pilon Leonardo; Piocho Diego; Pitzus Ariel; Polti Alejandro; Pozzer Domingo; Pozzer Leandro; Pozzer Pablo; Prados Graciela; Rasse Leandro; Retyk Enrique; Reyes Gabriela; Reyes Oscar; Ribotta Melissa; Ricon Daniel; Rios Natalia; Ritondale Noelia; Rivero Victor; Rojas Elirub; Rolon Jorge; Romano Alfredo; Salcedo Natalia; Sangiorgi Joaquin; Schapachnik Edgardo; Schidt Pablo; Schuster Maria; Secchi Jorge; Signh Marina; Soriano Lisandro; Taboada Carlos; Tapia Veronica; Tilca Antonio; Trotta Omar; Valentino Mariana; Vasquez Florencia; Velarde Mariscal Jose; Velasquez Raul; Villar Silvia; Votero Ezequiel; Zuñiga Silva Wilson*
